# Supplementary material for: Implementation of school-feeding programme and its effect on dietary adequacy among primary school children in Kisarawe district, Tanzania
Source: PLoS One. 2026 Aug 5;21(8):e0355391. doi: 10.1371/journal.pone.0355391 (PMC13440871; doi:10.1371/journal.pone.0355391)
Supplement: Supplementary file 3 [file pone.0355391.s004.docx]

Felistar Mwakasungura,

P.O. Box 65001,

MUHAS.

27^th^ March 2026.

To,

Academic Editor,

PLOS One.

**RE: RESPONSES TO REVIEWERS COMMENTS**

**Dear Sir/Madam,**

Refer to the heading above, kindly find the below responses from the comments we received on our manuscript submit to your esteemed journal.

**Response to reviewer 1**

1. The manuscript frequently uses "nutritional adequacy" and "dietary adequacy" interchangeably. In nutritional science, dietary adequacy (intake relative to requirements) is often considered a precursor to nutritional status (physiological outcome). I recommend consistently using terms like "dietary adequacy" when referring to intake (DDS and meal frequency) and reserving "undernutrition" for anthropometric or biochemical outcomes.

**Response**: Thank you for this feedback. We accept this recommendation. The term dietary adequacy, which refers to food intake (DDS and meal frequency), has been used to replace nutritional adequacy throughout the manuscript.

2. The study defines dietary diversity as consuming at least 4 out of 7 food groups. However, the current FAO/WHO standard for Minimum Dietary Diversity (MDD) for individuals (including adolescents) is typically validated at 5 out of 10 food groups to predict micronutrient adequacy. The authors should justify why the 4/7 threshold was chosen over the 5/10 standard.

**Response**: Thank you for this observation. We agree that FAO/WHO Minimum Dietary Diversity refers to consuming at least 5 out of 10 food groups. However, the threshold of consuming at least 4 out of 7 food groups for Minimum Dietary Diversity was used in this study because the Tanzanian National Guideline on School Feeding and Nutrition Services to Basic Education Students requires that schools provide at least 4 food groups to their students at a threshold. Adapting this standard in our study makes the study's outcomes and recommendations readily accessible and understandable to policymakers and stakeholders, thereby facilitating improvements in the country's school feeding services.

3. There is a critical contradiction regarding the study duration. The Abstract claims data collection occurred from "March to May 2025", while the Methods section specifies a four-day window from "22/4/2025 to 25/4/2025". The authors must clarify this and explain how 300 face-to-face interviews were conducted in four days, including details on the number of enumerators used to ensure data quality.

**Response**: Thank you for this observation. We agree on the need to clarify the data collection process in order to ensure feasibility and data quality.

The 300 face-to-face interviews were conducted over a period of four days using a team-based data collection approach. Five trained research assistants were engaged in the field. All enumerators had prior experience in data collection and received additional training specific to this study, including the interview techniques, ethical considerations, and administration of the questionnaire.

Data collection was conducted concurrently across the selected schools, with multiple enumerators deployed per site. On average:

i. Each enumerator conducted approximately 15 interviews per day
ii. Each interview lasted about 15–20 minutes, as it primarily involved structured questions (24-hour recall and questionnaire items)

To ensure data quality, the following measures were implemented:

i. All enumerators underwent training to ensure consistency in administering the tool.
ii. The questionnaire was piloted prior to data collection to refine clarity and flow.
iii. Field supervision was conducted by the principal investigator to monitor adherence to procedures.
iv. Data were collected using KOBO Collect on tablets, minimizing manual errors and enabling immediate validation checks.
v. Completed interviews were checked daily for completeness and consistency, and feedback was provided to enumerators where necessary.

This coordinated approach allowed efficient data collection within the limited timeframe while maintaining high data quality standards. We have now clarified these details in the Data Collection section line 110 to 127.

4. The study relies on a single 24-hour dietary recall, which cannot account for day-to-day variation. The authors should explicitly list this as a major limitation and discuss how it might have affected the results, particularly for the "marginal" significance found in dietary diversity.

**Response**: Thank you for this observation. We acknowledge that the use of a single 24-hour dietary recall does not capture day-to-day variation in individual dietary intake and may therefore not fully reflect daily dietary patterns. We have now included this as a limitation in the Discussion section (line 260). Relying on a single recall may lead to misclassification of dietary diversity status. This could have affected the observed association between participation in the school feeding program and dietary diversity.

In particular, we recognize that this limitation may partly explain the marginal statistical significance observed (APR = 1.45; p = 0.051), as random measurement error from day-to-day dietary variation may bias estimates toward the null.

5. The manuscript states that written consent was obtained from head teachers, while students provided only verbal assent. For minors (10-14 years), standard ethical practice requires informed written consent from parents or legal guardians in addition to student assent. The authors must clarify if parental consent was bypassed and provide a strong ethical justification if so.

**Response**: Thank you for this observation. Changes have been incorporated to further clarify the ethical procedures under Ethical approval, informed consent, and Patient Participation Section. Line 143 to 148.

6. The manuscript states that written consent was obtained from head teachers, while students provided only verbal assent. For minors (10-14 years), standard ethical practice requires informed written consent from parents or legal guardians in addition to student assent. The authors must clarify if parental consent was bypassed and provide a strong ethical justification if so.

**Response**: Thank you for this important comment. We agree that assessing comparability between participants and non-participants is important to evaluate potential confounding. While a separate comparative Table 1 was not presented, key baseline characteristics, including parental education, household size, and household food insecurity, were included in the multivariable regression models to control for confounding.

To improve clarity, we have now added a descriptive comparison of these characteristics by participation status in the Results section. This provides additional transparency regarding baseline differences between groups while maintaining a concise presentation. Kindly find this in line 177.

7. In the Results and Abstract, the p-value of 0.051 for dietary diversity is described as having "did not reach statistical significance". Refine this to describe the finding as "marginally significant" or a "notable trend," especially given the large effect size (45% higher likelihood) and the potential lack of power or the limitations of a single-day recall.

**Response**: Thank you for this observation. The description has been refined to use the term “marginally significant” under the Results section, line 181.

8. To improve the "Results" section, the authors should report which specific food groups were most frequently added by the school meal. For instance, did the school meal provide the "protein gap" (legumes/meat) that was missing from home diets? This would provide much-needed depth to the finding that 40% of schools provided meals with at least four food groups.

**Response**: Thank you for this observation. During data collection, the number of food groups provided by a school was documented through an observation checklist, which did not capture on the type of food. However, from the questionnaire, we can know the type of food that the children consumed in the previous 24 hours, and that has been added in the Results section, line 172 to 174.

9. The finding that males were 48% less likely to meet meal frequency requirements is significant. The discussion should be expanded to explore cultural reasons for this in rural Kisarawe, such as boys being more likely to be involved in livestock herding or agricultural labor that keeps them away from scheduled meal times.

**Response**: Thank you for this observation. Indeed, the discussion has been expanded to explore cultural reasons why males are less likely to meet meal frequency requirements. This may be found in Discussion section, line 257 to 262.

10. The study took place in April. The authors should discuss whether this was a "lean" or "harvest" season in Kisarawe and how this affects the generalizability of their dietary diversity scores.

**Response**: Thank you for this observation. The study took place in April which is a lean month in Kisarawe, this season is characterized by reduced food availability and increased reliance on markets which indeed may affect diversity scores, hence limiting the study generalizability to seasonal rather that year-round patterns. This has further been discussed under the Discussion section, line 230 to 234.

11. The manuscript should discuss the possibility of "home-meal substitution"—where parents reduce food portions at home because the child ate at school. This could explain why the school feeding program had a massive impact on meal frequency (12x higher likelihood) but only a marginal impact on overall dietary diversity.

**Response**: Thank you for this observation. We agree home meal substitution may be used to explain the results where school feeding program has massively contributed to increased meal frequency with only marginal change in food diversity. Parents may reduce portion sizes or omit meals at home once a child is perceived to have eaten at school, thereby increasing total meal frequency without necessarily improving the diversity of foods consumed across the day. This is further discussed under the Discussion section, line 272 to 284.

12. The finding that none of the schools fully met national guidelines due to lack of dining areas or storage is a major point. The authors should emphasize that the "effect" of the program is likely attenuated by these implementation failures.

**Response**: Thank you for this observation. We agree on the emphasis of this point and it has been added under the Conclusion section, line 301 to 302.

**Response to Reviewer 2**

1. Line 99 mentions “The exposure variable was participation in the school feeding program” and table 1 shows. Participation in school feeding program Yes 195 (65%); No 105 (35%). I am unclear as to how children attending the same school which had similar school feeding programs running were excluded from participation. Was this because some schools did not have the program (and therefore the children studying there naturally did not participate) or was it because of absenteeism of students the previous day?

**Response**: Thank you for this observation. The study found that while studying at a school that had a school feeding program not all students participated. This was because they were required to contribute a certain amount of money either weekly or everyday depending on the school’s arrangement, as part of parents’ support for the program. For many who did not participate regularly, the reason was that they were not provided with money at home.

2. Lines 152-154 mention “only 40% provided meals containing at least four different food groups as recommended. Despite the presence of school feeding programs, only 65% of enrolled students participated regularly due to economic barriers”. I am curious to know whether the researchers would also like to do some basic analysis related to the causes behind the consumption of four food groups or lack of it. While information is provided that 65% students participated in the school feeding program while 40% participants were provided four food groups in the meals served, it would be interesting to understand the relation between participation in the program, availability and food groups in the school and food groups consumed at home to the outcomes of the study.

**Response**: Thank you for this feedback. Yes, we will be glad to explore in future studies to understand the causes of consumption of four food groups or lack of it. However, based on the data we collected in this study, we did an analysis to understand the association between participating in school feeding and consumption of four food groups or not. May you kindly see it in the Results section, line 175 to 179.

Sincerely,

Felistar Mwakasungura, MD, MSc.
