# Supplementary material for: Implementation of school-feeding programme and its effect on dietary adequacy among primary school children in Kisarawe district, Tanzania
Source: PLoS One. 2026 Aug 5;21(8):e0355391. doi: 10.1371/journal.pone.0355391 (PMC13440871; doi:10.1371/journal.pone.0355391)
Supplement: Supplementary file 4 [file pone.0355391.s002.docx]

Reviewer’s queries

1. Line 99 mentions “The exposure variable was participation in the school feeding program” and table 1 shows Participation in school feeding program Yes 195 65%

No 105 35%.

I am unclear as to how children attending the same school which had similar school feeding programs running were excluded from participation. Was this because some schools did not have the program (and therefore the children studying there naturally did not participate) or was it because of absenteeism of students the previous day?

2. Lines 152-154 mention “only 40% provided meals containing at least four different food groups as recommended. Despite the presence of school feeding programs, only 65% of enrolled students participated regularly due to economic barriers”.

I am curious to know whether the researchers would also like to do some basic analysis related to the causes behind the consumption of four food groups or lack of it. While information is provided that 65% students participated in the school feeding program while 40% participants were provided four food groups in the meals served, it would be interesting to understand the relation between participation in the program, availability and food groups in the school and food groups consumed at home to the outcomes of the study.
