# Supplementary material for: Implementation of school-feeding programme and its effect on dietary adequacy among primary school children in Kisarawe district, Tanzania
Source: PLoS One. 2026 Aug 5;21(8):e0355391. doi: 10.1371/journal.pone.0355391 (PMC13440871; doi:10.1371/journal.pone.0355391)
Supplement: Peer review history

# Peer review: Decision Letter 0

**DOI:** 10.1371/journal.pone.0355391.r001

**Authors:** Stephen Dubik

13 Mar 2026

PONE-D-25-56115

Implementation of school-feeding programme and its effect on nutritional adequacy among primary school children in Kisarawe district, Tanzania.

PLOS One

Dear Dr. Mwakasungura,

Thank you for submitting your manuscript to PLOS ONE. After careful consideration, we feel that it has merit but does not fully meet PLOS ONE’s publication criteria as it currently stands. Therefore, we invite you to submit a revised version of the manuscript that addresses the points raised during the review process.

Please submit your revised manuscript by Apr 27 2026 11:59PM. If you will need more time than this to complete your revisions, please reply to this message or contact the journal office at plosone@plos.org. When you're ready to submit your revision, log on to https://www.editorialmanager.com/pone/ and select the 'Submissions Needing Revision' folder to locate your manuscript file.

Please include the following items when submitting your revised manuscript:

- A letter that responds to each point raised by the academic editor and reviewer(s). You should upload this letter as a separate file labeled 'Response to Reviewers'.
- A marked-up copy of your manuscript that highlights changes made to the original version. You should upload this as a separate file labeled 'Revised Manuscript with Track Changes'.
- An unmarked version of your revised paper without tracked changes. You should upload this as a separate file labeled 'Manuscript'.

If you would like to make changes to your financial disclosure, please include your updated statement in your cover letter. Guidelines for resubmitting your figure files are available below the reviewer comments at the end of this letter.

If applicable, we recommend that you deposit your laboratory protocols in protocols.io to enhance the reproducibility of your results. Protocols.io assigns your protocol its own identifier (DOI) so that it can be cited independently in the future. For instructions see: https://journals.plos.org/plosone/s/submission-guidelines#loc-laboratory-protocols. Additionally, PLOS ONE offers an option for publishing peer-reviewed Lab Protocol articles, which describe protocols hosted on protocols.io. Read more information on sharing protocols at https://plos.org/protocols?utm_medium=editorial-email&utm_source=authorletters&utm_campaign=protocols.

We look forward to receiving your revised manuscript.

Kind regards,

Stephen Dajaan Dubik, BSc, MPH, MPhil

Academic Editor

PLOS One

Journal Requirements:

When submitting your revision, we need you to address these additional requirements.

1. Please ensure that your manuscript meets PLOS ONE's style requirements, including those for file naming. The PLOS ONE style templates can be found at

https://journals.plos.org/plosone/s/file?id=wjVg/PLOSOne_formatting_sample_main_body.pdf and

https://journals.plos.org/plosone/s/file?id=ba62/PLOSOne_formatting_sample_title_authors_affiliations.pdf

2. We note you have included a table to which you do not refer in the text of your manuscript. Please ensure that you refer to Table 2 in your text; if accepted, production will need this reference to link the reader to the Table.

3. If the reviewer comments include a recommendation to cite specific previously published works, please review and evaluate these publications to determine whether they are relevant and should be cited. There is no requirement to cite these works unless the editor has indicated otherwise.

[Note: HTML markup is below. Please do not edit.]

Reviewers' comments:

Reviewer's Responses to Questions

**Comments to the Author**

1. Is the manuscript technically sound, and do the data support the conclusions?

The manuscript must describe a technically sound piece of scientific research with data that supports the conclusions. Experiments must have been conducted rigorously, with appropriate controls, replication, and sample sizes. The conclusions must be drawn appropriately based on the data presented.

Reviewer #1: No

Reviewer #2: Yes

**********

2. Has the statistical analysis been performed appropriately and rigorously?

Reviewer #1: No

Reviewer #2: Yes

**********

3. Have the authors made all data underlying the findings in their manuscript fully available?

The PLOS Data policy requires authors to make all data underlying the findings described in their manuscript fully available without restriction, with rare exception (please refer to the Data Availability Statement in the manuscript PDF file). The data should be provided as part of the manuscript or its supporting information, or deposited to a public repository. For example, in addition to summary statistics, the data points behind means, medians and variance measures should be available. If there are restrictions on publicly sharing data—e.g. participant privacy or use of data from a third party—those must be specified.

Reviewer #1: Yes

Reviewer #2: Yes

**********

4. Is the manuscript presented in an intelligible fashion and written in standard English?

PLOS ONE does not copyedit accepted manuscripts, so the language in submitted articles must be clear, correct, and unambiguous. Any typographical or grammatical errors should be corrected at revision, so please note any specific errors here.

Reviewer #1: No

Reviewer #2: Yes

**********

5. Review Comments to the Author

Please use the space provided to explain your answers to the questions above. You may also include additional comments for the author, including concerns about dual publication, research ethics, or publication ethics. (Please upload your review as an attachment if it exceeds 20,000 characters)

Reviewer #1: Title: Implementation of school-feeding programme and its effect on nutritional adequacy among primary school children in Kisarawe district, Tanzania.

Aim(s): To evaluate the implementation status and effects of school feeding programs on nutritional adequacy (measured via dietary diversity and meal frequency) among primary-school children in Kisarawe District, Tanzania.

This paper examined the cross-sectional associations between school feeding programs and dietary adequacy among primary school adolescents in Tanzanian Kisarawe district, where severe undernutrition is a pervasive phenomenon. The research question is important, not well covered by current research, and well-defined. Unfortunately, central constructs are ambiguously termed (e.g. nutritional adequacy/dietary adequacy, which I think are not interchangeable but represent two different stages of the health process) or not well operationalized (e.g. how is DDS determined). The language is hampering the quality of the manuscript and definitely needs correction and clarification. Central terms have to be defined and consequently labeled in accordance with the field.

I suggest the following major points:

1.) The manuscript frequently uses "nutritional adequacy" and "dietary adequacy" interchangeably. In nutritional science, dietary adequacy (intake relative to requirements) is often considered a precursor to nutritional status (physiological outcome). I recommend consistently using terms like "dietary adequacy" when referring to intake (DDS and meal frequency) and reserving "undernutrition" for anthropometric or biochemical outcomes.

2.) The study defines dietary diversity as consuming at least 4 out of 7 food groups. However, the current FAO/WHO standard for Minimum Dietary Diversity (MDD) for individuals (including adolescents) is typically validated at 5 out of 10 food groups to predict micronutrient adequacy. The authors should justify why the 4/7 threshold was chosen over the 5/10 standard.

3.) There is a critical contradiction regarding the study duration. The Abstract claims data collection occurred from "March to May 2025", while the Methods section specifies a four-day window from "22/4/2025 to 25/4/2025". The authors must clarify this and explain how 300 face-to-face interviews were conducted in four days, including details on the number of enumerators used to ensure data quality.

4.) The study relies on a single 24-hour dietary recall, which cannot account for day-to-day variation. The authors should explicitly list this as a major limitation and discuss how it might have affected the results, particularly for the "marginal" significance found in dietary diversity.

5.) The manuscript states that written consent was obtained from head teachers, while students provided only verbal assent. For minors (10-14 years), standard ethical practice requires informed written consent from parents or legal guardians in addition to student assent. The authors must clarify if parental consent was bypassed and provide a strong ethical justification if so.

6.) Baseline Balance (Table 1): While the manuscript includes a Table 1 for general characteristics, it fails to provide a comparative "Table 1" that shows if the participating and non-participating groups were balanced at baseline regarding socio-economic status (SES), parental education, or household food insecurity. Without this, the associations found in the regression could be confounded by pre-existing household differences.

7.) In the Results and Abstract, the p-value of 0.051 for dietary diversity is described as having "did not reach statistical significance". Refine this to describe the finding as "marginally significant" or a "notable trend," especially given the large effect size (45% higher likelihood) and the potential lack of power or the limitations of a single-day recall.

8.) To improve the "Results" section, the authors should report which specific food groups were most frequently added by the school meal. For instance, did the school meal provide the "protein gap" (legumes/meat) that was missing from home diets? This would provide much-needed depth to the finding that 40% of schools provided meals with at least four food groups.

9.) The finding that males were 48% less likely to meet meal frequency requirements is significant. The discussion should be expanded to explore cultural reasons for this in rural Kisarawe, such as boys being more likely to be involved in livestock herding or agricultural labor that keeps them away from scheduled meal times.

10.) The study took place in April. The authors should discuss whether this was a "lean" or "harvest" season in Kisarawe and how this affects the generalizability of their dietary diversity scores.

11.) The manuscript should discuss the possibility of "home-meal substitution"—where parents reduce food portions at home because the child ate at school. This could explain why the school feeding program had a massive impact on meal frequency (12x higher likelihood) but only a marginal impact on overall dietary diversity.

12.) The finding that none of the schools fully met national guidelines due to lack of dining areas or storage is a major point. The authors should emphasize that the "effect" of the program is likely attenuated by these implementation failures.

Reviewer #2: 1. Line 99 mentions “The exposure variable was participation in the school feeding program” and table 1 shows Participation in school feeding program Yes 195 65%

No 105 35%.

I am unclear as to how children attending the same school which had similar school feeding programs running were excluded from participation. Was this because some schools did not have the program (and therefore the children studying there naturally did not participate) or was it because of absenteeism of students the previous day?

2. Lines 152-154 mention “only 40% provided meals containing at least four different food groups as recommended. Despite the presence of school feeding programs, only 65% of enrolled students participated regularly due to economic barriers”.

I am curious to know whether the researchers would also like to do some basic analysis related to the causes behind the consumption of four food groups or lack of it. While information is provided that 65% students participated in the school feeding program while 40% participants were provided four food groups in the meals served, it would be interesting to understand the relation between participation in the program, availability and food groups in the school and food groups consumed at home to the outcomes of the study.

**********

6. PLOS authors have the option to publish the peer review history of their article (what does this mean?). If published, this will include your full peer review and any attached files.

If you choose “no”, your identity will remain anonymous but your review may still be made public.

**Do you want your identity to be public for this peer review?**  For information about this choice, including consent withdrawal, please see our Privacy Policy.

Reviewer #1: **Yes:**  Belete Yimer

Reviewer #2: No

**********

[NOTE: If reviewer comments were submitted as an attachment file, they will be attached to this email and accessible via the submission site. Please log into your account, locate the manuscript record, and check for the action link "View Attachments". If this link does not appear, there are no attachment files.]

To ensure your figures meet our technical requirements, please review our figure guidelines: https://journals.plos.org/plosone/s/figures

You may also use PLOS’s free figure tool, NAAS, to help you prepare publication quality figures: https://journals.plos.org/plosone/s/figures#loc-tools-for-figure-preparation.

NAAS will assess whether your figures meet our technical requirements by comparing each figure against our figure specifications.

Attachment Submitted filename: review.docx

# Author comment: Author response to Decision Letter 1

**DOI:** 10.1371/journal.pone.0355391.r002

11 Apr 2026

Response to reviewer 1

1. The manuscript frequently uses "nutritional adequacy" and "dietary adequacy" interchangeably. In nutritional science, dietary adequacy (intake relative to requirements) is often considered a precursor to nutritional status (physiological outcome). I recommend consistently using terms like "dietary adequacy" when referring to intake (DDS and meal frequency) and reserving "undernutrition" for anthropometric or biochemical outcomes.

Response: Thank you for this feedback. We accept this recommendation. The term dietary adequacy, which refers to food intake (DDS and meal frequency), has been used to replace nutritional adequacy throughout the manuscript.

2. The study defines dietary diversity as consuming at least 4 out of 7 food groups. However, the current FAO/WHO standard for Minimum Dietary Diversity (MDD) for individuals (including adolescents) is typically validated at 5 out of 10 food groups to predict micronutrient adequacy. The authors should justify why the 4/7 threshold was chosen over the 5/10 standard.

Response: Thank you for this observation. We agree that FAO/WHO Minimum Dietary Diversity refers to consuming at least 5 out of 10 food groups. However, the threshold of consuming at least 4 out of 7 food groups for Minimum Dietary Diversity was used in this study because the Tanzanian National Guideline on School Feeding and Nutrition Services to Basic Education Students requires that schools provide at least 4 food groups to their students at a threshold. Adapting this standard in our study makes the study's outcomes and recommendations readily accessible and understandable to policymakers and stakeholders, thereby facilitating improvements in the country's school feeding services.

3. There is a critical contradiction regarding the study duration. The Abstract claims data collection occurred from "March to May 2025", while the Methods section specifies a four-day window from "22/4/2025 to 25/4/2025". The authors must clarify this and explain how 300 face-to-face interviews were conducted in four days, including details on the number of enumerators used to ensure data quality.

Response: Thank you for this observation. We agree on the need to clarify the data collection process in order to ensure feasibility and data quality.

The 300 face-to-face interviews were conducted over a period of four days using a team-based data collection approach. Five trained research assistants were engaged in the field. All enumerators had prior experience in data collection and received additional training specific to this study, including the interview techniques, ethical considerations, and administration of the questionnaire.

Data collection was conducted concurrently across the selected schools, with multiple enumerators deployed per site. On average:

i. Each enumerator conducted approximately 15 interviews per day

ii. Each interview lasted about 15–20 minutes, as it primarily involved structured questions (24-hour recall and questionnaire items)

To ensure data quality, the following measures were implemented:

i. All enumerators underwent training to ensure consistency in administering the tool.

ii. The questionnaire was piloted prior to data collection to refine clarity and flow.

iii. Field supervision was conducted by the principal investigator to monitor adherence to procedures.

iv. Data were collected using KOBO Collect on tablets, minimizing manual errors and enabling immediate validation checks.

v. Completed interviews were checked daily for completeness and consistency, and feedback was provided to enumerators where necessary.

This coordinated approach allowed efficient data collection within the limited timeframe while maintaining high data quality standards. We have now clarified these details in the Data Collection section line 110 to 127.

4. The study relies on a single 24-hour dietary recall, which cannot account for day-to-day variation. The authors should explicitly list this as a major limitation and discuss how it might have affected the results, particularly for the "marginal" significance found in dietary diversity.

Response: Thank you for this observation. We acknowledge that the use of a single 24-hour dietary recall does not capture day-to-day variation in individual dietary intake and may therefore not fully reflect daily dietary patterns. We have now included this as a limitation in the Discussion section (line 260). Relying on a single recall may lead to misclassification of dietary diversity status. This could have affected the observed association between participation in the school feeding program and dietary diversity.

In particular, we recognize that this limitation may partly explain the marginal statistical significance observed (APR = 1.45; p = 0.051), as random measurement error from day-to-day dietary variation may bias estimates toward the null.

5. The manuscript states that written consent was obtained from head teachers, while students provided only verbal assent. For minors (10-14 years), standard ethical practice requires informed written consent from parents or legal guardians in addition to student assent. The authors must clarify if parental consent was bypassed and provide a strong ethical justification if so.

Response: Thank you for this observation. Changes have been incorporated to further clarify the ethical procedures under Ethical approval, informed consent, and Patient Participation Section. Line 143 to 148.

6. The manuscript states that written consent was obtained from head teachers, while students provided only verbal assent. For minors (10-14 years), standard ethical practice requires informed written consent from parents or legal guardians in addition to student assent. The authors must clarify if parental consent was bypassed and provide a strong ethical justification if so.

Response: Thank you for this important comment. We agree that assessing comparability between participants and non-participants is important to evaluate potential confounding. While a separate comparative Table 1 was not presented, key baseline characteristics, including parental education, household size, and household food insecurity, were included in the multivariable regression models to control for confounding.

To improve clarity, we have now added a descriptive comparison of these characteristics by participation status in the Results section. This provides additional transparency regarding baseline differences between groups while maintaining a concise presentation. Kindly find this in line 177.

7. In the Results and Abstract, the p-value of 0.051 for dietary diversity is described as having "did not reach statistical significance". Refine this to describe the finding as "marginally significant" or a "notable trend," especially given the large effect size (45% higher likelihood) and the potential lack of power or the limitations of a single-day recall.

Response: Thank you for this observation. The description has been refined to use the term “marginally significant” under the Results section, line 181.

8. To improve the "Results" section, the authors should report which specific food groups were most frequently added by the school meal. For instance, did the school meal provide the "protein gap" (legumes/meat) that was missing from home diets? This would provide much-needed depth to the finding that 40% of schools provided meals with at least four food groups.

Response: Thank you for this observation. During data collection, the number of food groups provided by a school was documented through an observation checklist, which did not capture on the type of food. However, from the questionnaire, we can know the type of food that the children consumed in the previous 24 hours, and that has been added in the Results section, line 172 to 174.

9. The finding that males were 48% less likely to meet meal frequency requirements is significant. The discussion should be expanded to explore cultural reasons for this in rural Kisarawe, such as boys being more likely to be involved in livestock herding or agricultural labor that keeps them away from scheduled meal times.

Response: Thank you for this observation. Indeed, the discussion has been expanded to explore cultural reasons why males are less likely to meet meal frequency requirements. This may be found in Discussion section, line 257 to 262.

10. The study took place in April. The authors should discuss whether this was a "lean" or "harvest" season in Kisarawe and how this affects the generalizability of their dietary diversity scores.

Response: Thank you for this observation. The study took place in April which is a lean month in Kisarawe, this season is characterized by reduced food availability and increased reliance on markets which indeed may affect diversity scores, hence limiting the study generalizability to seasonal rather that year-round patterns. This has further been discussed under the Discussion section, line 230 to 234.

11. The manuscript should discuss the possibility of "home-meal substitution"—where parents reduce food portions at home because the child ate at school. This could explain why the school feeding program had a massive impact on meal frequency (12x higher likelihood) but only a marginal impact on overall dietary diversity.

Response: Thank you for this observation. We agree home meal substitution may be used to explain the results where school feeding program has massively contributed to increased meal frequency with only marginal change in food diversity. Parents may reduce portion sizes or omit meals at home once a child is perceived to have eaten at school, thereby increasing total meal frequency without necessarily improving the diversity of foods consumed across the day. This is further discussed under the Discussion section, line 272 to 284.

12. The finding that none of the schools fully met national guidelines due to lack of dining areas or storage is a major point. The authors should emphasize that the "effect" of the program is likely attenuated by these implementation failures.

Response: Thank you for this observation. We agree on the emphasis of this point and it has been added under the Conclusion section, line 301 to 302.

Response to Reviewer 2

1. Line 99 mentions “The exposure variable was participation in the school feeding program” and table 1 shows. Participation in school feeding program Yes 195 (65%); No 105 (35%). I am unclear as to how children attending the same school which had similar school feeding programs running were excluded from participation. Was this because some schools did not have the program (and therefore the children studying there naturally did not participate) or was it because of absenteeism of students the previous day?

Response: Thank you for this observation. The study found that while studying at a school that had a school feeding program not all students participated. This was because they were required to contribute a certain amount of money either weekly or everyday depending on the school’s arrangement, as part of parents’ support for the program. For many who did not participate regularly, the reason was that they were not provided with money at home.

2. Lines 152-154 mention “only 40% provided meals containing at least four different food groups as recommended. Despite the presence of school feeding programs, only 65% of enrolled students participated regularly due to economic barriers”. I am curious to know whether the researchers would also like to do some basic analysis related to the causes behind the consumption of four food groups or lack of it. While information is provided that 65% students participated in the school feeding program while 40% participants were provided four food groups in the meals served, it would be interesting to understand the relation between participation in the program, availability and food groups in the school and food groups consumed at home to the outcomes of the study.

Response: Thank you for this feedback. Yes, we will be glad to explore in future studies to understand the causes of consumption of four food groups or lack of it. However, based on the data we collected in this study, we did an analysis to understand the association between participating in school feeding and consumption of four food groups or not. May you kindly see it in the Results section, line 175 to 179.

Attachment Submitted filename: Response to Reviewers.docx

# Peer review: Decision Letter 1

**DOI:** 10.1371/journal.pone.0355391.r003

**Authors:** Stephen Dubik

21 Jul 2026

Implementation of school-feeding programme and its effect on dietary adequacy among primary school children in Kisarawe district, Tanzania.

PONE-D-25-56115R1

Dear Dr. Felistar William,

We’re pleased to inform you that your manuscript has been judged scientifically suitable for publication and will be formally accepted for publication once it meets all outstanding technical requirements.

Within one week, you’ll receive an e-mail detailing the required amendments. When these have been addressed, you’ll receive a formal acceptance letter and your manuscript will be scheduled for publication.

An invoice will be generated when your article is formally accepted. Please note, if your institution has a publishing partnership with PLOS and your article meets the relevant criteria, all or part of your publication costs will be covered. Please make sure your user information is up-to-date by logging into Editorial Manager at Editorial Manager® and clicking the ‘Update My Information' link at the top of the page. For questions related to billing, please contact billing support.

If your institution or institutions have a press office, please notify them about your upcoming paper to help maximize its impact. If they’ll be preparing press materials, please inform our press team as soon as possible -- no later than 48 hours after receiving the formal acceptance. Your manuscript will remain under strict press embargo until 2 pm Eastern Time on the date of publication. For more information, please contact onepress@plos.org.

Kind regards,

Stephen Dajaan Dubik, BSc, MPH, MPhil

Academic Editor

PLOS One

Additional Editor Comments (optional):

Reviewers' comments:

Reviewer's Responses to Questions

**Comments to the Author**

1. If the authors have adequately addressed your comments raised in a previous round of review and you feel that this manuscript is now acceptable for publication, you may indicate that here to bypass the “Comments to the Author” section, enter your conflict of interest statement in the “Confidential to Editor” section, and submit your "Accept" recommendation.

Reviewer #2: All comments have been addressed

**********

2. Is the manuscript technically sound, and do the data support the conclusions?

The manuscript must describe a technically sound piece of scientific research with data that supports the conclusions. Experiments must have been conducted rigorously, with appropriate controls, replication, and sample sizes. The conclusions must be drawn appropriately based on the data presented.

Reviewer #2: Yes

**********

3. Has the statistical analysis been performed appropriately and rigorously?

Reviewer #2: Yes

**********

4. Have the authors made all data underlying the findings in their manuscript fully available?

The PLOS Data policy requires authors to make all data underlying the findings described in their manuscript fully available without restriction, with rare exception (please refer to the Data Availability Statement in the manuscript PDF file). The data should be provided as part of the manuscript or its supporting information, or deposited to a public repository. For example, in addition to summary statistics, the data points behind means, medians and variance measures should be available. If there are restrictions on publicly sharing data—e.g. participant privacy or use of data from a third party—those must be specified.

Reviewer #2: Yes

**********

5. Is the manuscript presented in an intelligible fashion and written in standard English?

PLOS ONE does not copyedit accepted manuscripts, so the language in submitted articles must be clear, correct, and unambiguous. Any typographical or grammatical errors should be corrected at revision, so please note any specific errors here.

Reviewer #2: Yes

**********

6. Review Comments to the Author

Please use the space provided to explain your answers to the questions above. You may also include additional comments for the author, including concerns about dual publication, research ethics, or publication ethics. (Please upload your review as an attachment if it exceeds 20,000 characters)

Reviewer #2: (No Response)

**********

7. PLOS authors have the option to publish the peer review history of their article (what does this mean?). If published, this will include your full peer review and any attached files.

If you choose “no”, your identity will remain anonymous but your review may still be made public.

**Do you want your identity to be public for this peer review?**  For information about this choice, including consent withdrawal, please see our Privacy Policy.

Reviewer #2: **Yes:**  Dr Narottam Pradhan

**********

# Editor report: Acceptance letter

**DOI:** 10.1371/journal.pone.0355391.r004

**Authors:** Stephen Dubik

PONE-D-25-56115R1

PLOS One

Dear Dr. Mwakasungura,

I'm pleased to inform you that your manuscript has been deemed suitable for publication in PLOS One. Congratulations! Your manuscript is now being handed over to our production team.

At this stage, our production department will prepare your paper for publication. This includes ensuring the following:

* All references, tables, and figures are properly cited

* All relevant supporting information is included in the manuscript submission,

* There are no issues that prevent the paper from being properly typeset

You will receive further instructions from the production team, including instructions on how to review your proof when it is ready. Please keep in mind that we are working through a large volume of accepted articles, so please give us a few days to review your paper and let you know the next and final steps.

Lastly, if your institution or institutions have a press office, please let them know about your upcoming paper now to help maximize its impact. If they'll be preparing press materials, please inform our press team within the next 48 hours. Your manuscript will remain under strict press embargo until 2 pm Eastern Time on the date of publication. For more information, please contact onepress@plos.org.

You will receive an invoice from PLOS for your publication fee after your manuscript has reached the completed accept phase. If you receive an email requesting payment before acceptance or for any other service, this may be a phishing scheme. Learn how to identify phishing emails and protect your accounts at https://explore.plos.org/phishing.

If we can help with anything else, please email us at customercare@plos.org.

Thank you for submitting your work to PLOS ONE and supporting open access.

Kind regards,

PLOS ONE Editorial Office Staff

on behalf of

Dr. Stephen Dajaan Dubik

Academic Editor

PLOS One
